# Age-Related Differences in Visual Attention to Heritage Tourism: An Eye-Tracking Study

**DOI:** 10.3390/jemr18030016

**Published:** 2025-05-08

**Authors:** Linlin Yuan, Zihao Cao, Yongchun Mao, Mohd Hafizal Mohd Isa, Muhammad Hafeez Abdul Nasir

**Affiliations:** 1 School of Housing, Building & Planning, Universiti Sains Malaysia, George Town 11800, Malaysia; yuanlinlin@student.usm.my; 2 School of the Arts, Universiti Sains Malaysia, George Town 11800, Malaysia; caozihao@student.usm.my; 3 School of Distance Education, Universiti Sains Malaysia, George Town 11800, Malaysia; 4 Faculty of Built Environment, Universiti Malaya, Kuala Lumpur 50603, Malaysia; hafizal.isa@um.edu.my

**Keywords:** heritage tourism, eye tracking, age differences, information processing, heritage marketing

## Abstract

With the rising significance of visual marketing, differences in how tourists from various age groups visually engage with tourism promotional materials remain insufficiently studied. This study recruited 48 participants and used a quasi-experimental design combined with eye-tracking technology to examine visual attention, scan path patterns, and their relationship to reading performance among different age groups. Independent t-tests, correlation analyses, and Lag Sequential Analysis were conducted to compare the differences between the two groups. Results indicated that elder participants had significantly higher fixation counts and longer fixation durations in text regions than younger participants, as well as higher perceived novelty scores. A positive correlation emerged between text fixation duration and perceived novelty. Additionally, elder participants showed greater interaction between text and images, while younger participants exhibited a more linear reading pattern. This study offers empirical insights to optimize tourism promotional materials, highlighting the need for age-specific communication strategies.

## 1. Introduction

In recent years, technological advancements and diverse tourist demands have amplified the importance of visual marketing in the tourism industry [1]. In promoting architectural heritage sites, visually rich materials not only highlight the destination’s aesthetic appeal but also communicate its historical and cultural significance. Brochures remain a traditional yet effective tool in tourism marketing, serving to attract offline visitors and provide information [2]. Such brochures typically integrate text and images to offer a comprehensive view of attractions, architectural features, and cultural context, aiding visitors in understanding and appreciating the destination’s appeal [3]. However, as visual material design evolves, the allocation of tourists’ visual attention, particularly during transitions between overall and detailed site information, warrants further investigation.

### 1.1. Tourism Marketing and Age Differences

Tourism marketing encompasses strategic communication and experiential components aimed at attracting and retaining tourists while supporting broader organizational objectives. The internet’s development has profoundly transformed tourism marketing, reshaping consumer behavior and preferences on digital platforms [4]. Nevertheless, traditional tourism marketing remains valuable, especially in contexts emphasizing cultural and experiential aspects [5]. In the tourism industry, organizations employ marketing-oriented strategies to address consumer needs and achieve economic goals, prioritizing customer satisfaction and competitive positioning as integral to their business culture [6]. This dual nature of tourism marketing highlights the necessity of integrating online and traditional methods to effectively engage and attract diverse consumer demographics. For instance, widely used scenic spot brochures can be customized to cater to different consumer demographics [7].

Previous studies have extensively investigated age-related differences in tourism marketing [8,9,10]. For example, age differences in tourism booking habits affect the preference for traditional marketing channels: younger tourists favor online options, whereas elder groups are more likely to choose in-person bookings [8]. Furthermore, elder consumers, compared to younger ones, display unique preferences, motivations, and consumption patterns, requiring tailored marketing strategies to effectively target elder demographics in tourism [11]. However, most of these studies concentrate on post-purchase management and do not thoroughly examine how potential tourists from different age groups experience immediate perceptual differences during tourism decision-making. Consequently, limited attention has been given to the real-time perceptual feedback of potential tourists during the processing of tourism marketing content.

### 1.2. Visual Behavior and Perceived Novelty

Research on visual behavior provides valuable insights into how visual information is processed when engaging with tourism promotional materials, especially brochures integrating images and text. Tourism brochures typically use a multi-layered structure, progressing from an overall view to individual examples and detailed features, effectively guiding readers’ attention and comprehension of attractions [12]. Previous research has demonstrated that incorporating more images and employing list formats in promotional materials significantly affects readers’ visual attention and information processing [3]. Visual stimuli in tourism marketing evoke both conscious and unconscious psychological responses, which subsequently influence behavioral intentions. However, the mechanisms by which these psychological responses translate into intentions vary, underscoring the complexity of visual effects on consumer behavior [13,14].

Eye-tracking metrics offer valuable insights into how individuals allocate visual attention during information processing. Fixations and saccades represent two fundamental components analyzed in eye-tracking studies on visual behavior [15]. Fixations refer to relatively stable periods when the eye focuses on a specific area, whereas saccades denote movements between consecutive fixations, creating a scan path [16]. Eye-tracking studies employ metrics such as Fixation Count (FC), which quantifies the frequency of gaze stabilization on a specific location [17], and Fixation Duration (FD), which measures the time spent focusing on a particular area [18]. These metrics are critical for analyzing visual behavior and information processing during reading tasks [19]. Eye-tracking metrics are also intricately linked to broader cognitive processes, including recall and perception [20]. Event perception entails segmenting ongoing experiences into meaningful events, whereas event recall reconstructs scenes based on past experiences or imagined scenarios [21].

Think Aloud is a technique in which participants verbalize their thoughts during tasks, providing insights into their information processing [22]. This method is widely applied in educational research [23], generating explicit data on cognitive strategies utilized during reading tasks [24]. While Think Aloud highlights deep understanding, recall emphasizes the capacity to retrieve information from memory [25]. Perceived novelty enhances emotional experiences before a trip, contributing to positive outcomes [26]. However, individuals’ attraction to novelty is shaped by perceived outcomes; over time, preferences often shift from novel and exciting options to more familiar ones. This shift toward familiarity arises from a desire for personally meaningful conclusions [27]. Therefore, examining perceived novelty alongside recall measures, which indicate familiarity with specific elements, provides a more comprehensive insight into individuals’ travel intentions.

### 1.3. Research Foundation and Research Questions

Information processing theory explains how participants encode and recall visual stimuli, such as images and text, and how these processes contribute to the generation of perceived novelty [28,29,30]. These metrics identify the most salient or memorable stimulus elements, providing insights into the cognitive mechanisms underlying participants’ judgments and decisions [31]. Eye-tracking technology enables researchers to examine how tourists allocate visual attention across various levels of visual information, particularly in transitions between overall landscapes, buildings, and decorative details. As familiarity with promotional materials grows, tourists’ perception of novelty becomes a significant factor in shaping their travel motivations, boosting their interest and intention to explore new destinations [27,32].

Additionally, Involvement Theory [33] offers a strong theoretical foundation for analyzing the visual behavior of potential tourists when engaging with tourism promotional materials. This theory posits that individuals’ level of involvement with information shapes their processing strategies [34]. In tourism brochures, the layered integration of text and images—spanning overall destination views, individual buildings, and detailed decorations—elicits diverse psychological involvement responses from potential tourists.

This exploratory study utilizes eye-tracking techniques to gather visual data from two age groups of potential tourists reading brochures about architectural heritage scenic spots and correlates these data with self-reported reading task outcomes. Specifically, the study addresses the following research questions:

RQ1. Are there significant differences in reading performance and visual attention between younger and elder groups?
RQ2. What are the relationships between potential tourists’ age, visual attention distribution, and reading task outcomes?
RQ3. What are the visual behavior patterns of younger and elder potential tourists when reading scenic spot brochures?

We hypothesize that eye-tracking data and self-reported outcomes will capture diverse aspects of potential tourists’ pre-travel visual behavior and psychological perceptions. Significant differences in visual attention and reading outcomes are anticipated between the two groups, alongside interrelations among age, visual attention distribution, and reading task outcomes. Furthermore, younger and elder potential tourists are expected to demonstrate distinct visual transition patterns when reading the scenic spot brochure.

## 2. Materials and Methods

**Figure 1:**
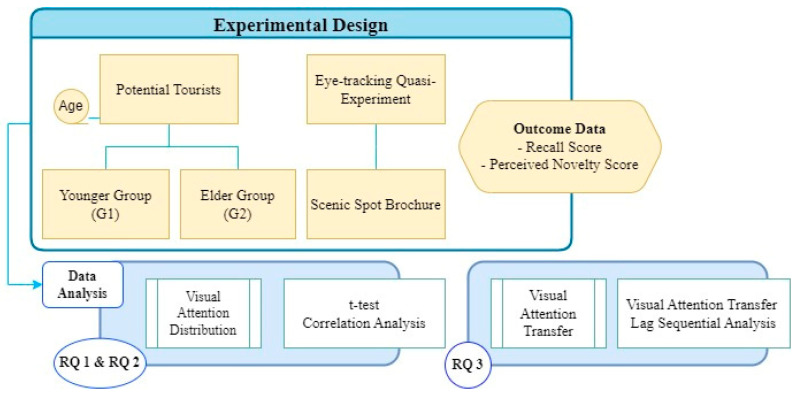
Research Framework.

To address the research questions, this study implemented a quasi-experimental design (Figure 1). Reading task outcomes were quantified using eye-tracking technology and questionnaires, then analyzed statistically to explore relationships among potential travelers’ age (younger and elder groups), think-aloud measures (recall and perceived novelty), and visual attention metrics (fixation count and reading time). Furthermore, Lag Sequential Analysis (LSA) was applied to compare the visual transition patterns of younger and elder participants while reading a scenic spot brochure.

Participants were instructed to read a scenic spot brochure featuring both text and images. They were assigned specific reading tasks to thoroughly comprehend the brochure’s information. The reading process lasted about 8 min, during which eye-tracking devices accurately recorded participants’ visual attention distribution and scan path patterns. After reading, participants completed a series of written questions related to the brochure content, taking approximately 10 min. Including equipment calibration and oral instructions provided by researchers, the data collection process for each participant took about 30 min. The research project was conducted from 10 July 2024 to 19 December 2024, covering critical stages such as participant recruitment, screening, and data collection.

### 2.1. Research Context and Stimuli

This study chose Hongcun, a UNESCO World Heritage Site, as its research context. Situated in Yi County, Huangshan City, Anhui Province, Hongcun was established during the Song Dynasty and boasts a history of over 800 years. Hongcun exemplifies Hui-style architecture, preserving numerous ancient buildings from the Ming and Qing Dynasties with well-maintained structures and layouts. In 2000, Hongcun was listed as a UNESCO World Heritage Site [35]. The village’s public and residential buildings showcase intricate wood, brick, and stone carvings, reflecting exceptional artistic value [36]. Attracting millions of visitors annually, Hongcun serves as a prominent cultural heritage destination in China [37] and offers credible tourism content for this study.

The stimulus material for this study consisted of a professionally reviewed scenic spot brochure commonly used in tourism marketing. The brochure was displayed as an image on a monitor with a resolution of 2365 × 2796 pixels. The brochure featured a single page with 490 Chinese characters and three images, accompanied by an English translation in Appendix A. All text content was derived from government websites, news outlets, and magazines. Six Areas of Interest (AOIs) were defined within the brochure to analyze participants’ visual attention distribution during reading: AOI 1 (1200 × 670 pixels): Introduction to the Architectural Heritage Site (all text); AOI 2 (750 × 670 pixels): one picture of the Architectural Heritage Site; AOI 3 (1200 × 670 pixels): Introduction to a Single Architectural Heritage (all text); AOI 4 (750 × 670 pixels): one picture of a Single Architectural Heritage; AOI 5 (1200 × 670 pixels): Introduction to Architectural Ornamental Details (all text); AOI 6 (750 × 670 pixels): one picture of Architectural Ornamental Details.

**Figure 2:**
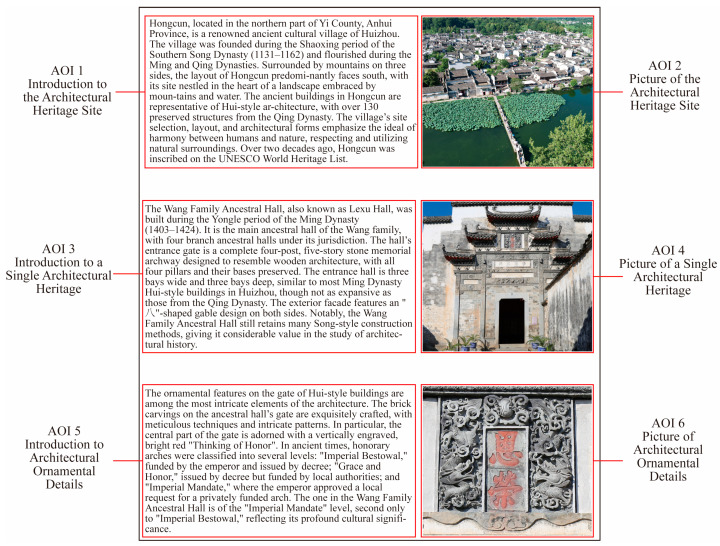
Designed stimuli (Displayed are images translated from English for ease of understanding. The author’s own design).

Figure 2 illustrates the layout and arrangement of these AOIs in the brochure. In this study, the AOIs were divided into text and image regions to better understand participants’ visual attention distribution and how they interact with different types of content while browsing the brochure. The cognitive and visual processing mechanisms for text and image information differ between younger and older participants, which can be reflected in the eye-tracking results. AOI 1 presents the geographic information and main features of the destination. AOI 3 emphasizes the key content and significance of a representative building. AOI 5 details the decorative aspects of the building. AOIs 2, 4, and 6 correspond to images on the right, paired with textual descriptions on the left.

The red borders and labels of the AOIs were invisible to participants during the reading task; they viewed only the complete brochure with integrated text and images.

### 2.2. Participants

A total of 59 eligible participants were recruited from the membership lists of four commercial travel agencies in Jinan City, with individuals holding tourism-related professional backgrounds excluded. Their native language is Chinese, and they have a good understanding of Chinese reading comprehension. Each participant completed a demographic questionnaire, enabling classification into younger and elder groups. Travel agency members were regarded as potential customers for future tourism product marketing. During recruitment, eight participants with prior visits to Hongcun and three with professional tourism expertise were excluded to mitigate biases from prior experience or specialized knowledge, which could affect visual cognitive performance [38]. This approach sought to capture broader cognitive responses to visual stimuli, thereby improving the external validity of the study results [39].

During data collection, participants with eye-tracking sampling rates below 90% were excluded, yielding a final dataset of 48 valid participants. The sample consisted of 25 males and 23 females, with 56.25% aged 18–25 years categorized as the younger group and 43.75% aged 45–55 years categorized as the elder group. All participants had normal or corrected vision ranging from 1.0 to 1.5, with no color blindness or visual impairments, ensuring that visual health factors did not affect the experiment. All participants gave written informed consent prior to participating in any tasks.

### 2.3. Measurement

Before the experiment commenced, participants were provided with a detailed explanation of the research procedure to ensure a thorough understanding, thereby enhancing the study’s reliability and validity. After providing written informed consent, participants entered the laboratory to acclimate to the environment. Researchers assisted participants in donning the eye-tracking device and performed calibration. Calibration, a critical step in eye-tracking experiments, adjusts the equipment to align with the individual’s eye physiology, ensuring accurate data that reflect visual attention [40]. During the process, researchers withheld specific details about the experimental stimuli, instructing participants only to keep their heads stationary while freely viewing the provided images.

**Figure 3:**
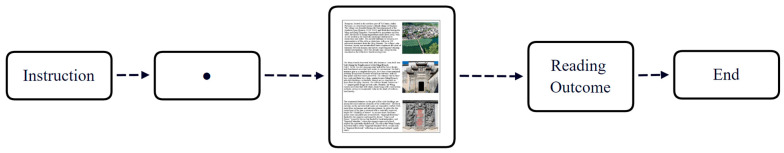
Experimental Process.

Eye movement data were gathered using the Tobii Pro Glasses 2 eye tracker (Tobii, Stockholm, Sweden). Previous research has shown that eye-tracking technology is an effective quantitative tool for visual studies, capable of capturing participants’ focus and illustrating their attention during observation [41]. Stimuli were presented on a 27-inch flat-screen LCD monitor with a resolution of 3840 × 2160 pixels and a refresh rate of 160 Hz. The experimental procedure, as shown in Figure 3, required participants to sit in front of the screen and read the displayed instructions. After confirming their understanding by pressing the space bar, a central fixation point was displayed on the screen for 1000 ms, followed by the automatic presentation of the experimental stimuli [40]. Participants pressed the space bar after viewing the first stimulus image to conclude that phase of the experiment. Standardizing event sequences and participant interactions with the stimuli minimized potential confounding variables and enhanced the reliability of subsequent analyses.

To evaluate participants’ visual attention to various Areas of Interest (AOIs) within the stimuli, two eye-tracking metrics were utilized: Fixation Count (FC) and Fixation Duration (FD). Detailed descriptions of these metrics are presented in Table 1 [42,43]. These metrics form the foundation for quantitative analysis, revealing how participants distribute their overall visual attention during reading. FC and FD are indicators used to measure the level of visual attention of participants [44]. FC refers to the number of times an individual’s visual attention shifts to a specific AOI, with a higher FC value indicating that the information in that area requires more references or is more engaging to the participant [17,45]. FD, on the other hand, refers to the duration of visual attention participants spend on a specific AOI, reflecting the richness of information in that area or the difficulty of information decoding [15,18]. By combining these two indicators, in this study, we can explain how younger and older participants allocate their visual attention when facing a tourism brochure, and we further discuss how they distribute their cognitive resources between images and text.

After the eye-tracking session, all participants answered the same questions and wrote their responses on blank paper. To evaluate participants’ information processing of the scenic spot, they were instructed to write a brief conclusion based on the brochure they had just read. Specifically, participants responded to two questions: (1) “Please write down your impressions of Hongcun based on the material you just read”, and (2) “Please describe what you found novel about Hongcun based on the material you just read”. The written responses were analyzed to examine the type and quantity of recalled content and how participants perceived novelty in the text and images. Written summaries were employed instead of oral responses to improve data validity and the reliability of cognitive measures, as they more accurately reflect participants’ thoughts and cognitive structures [46]. Given the convenience of handwriting compared to typing on a computer, participants were provided with paper for their responses. Participants were given unlimited time to digest and reflect on the material, though most completed the task within 10 min.

### 2.4. Data Analysis

Statistical analyses were performed on questionnaire scores and eye-tracking metrics within the Areas of Interest (AOIs), including Fixation Count (FC) and Fixation Duration (FD). The mean and variance were calculated for each metric to ensure precision. Eye-tracking data were collected using the Tobii Pro Glasses Controller (Tobii Pro, Stockholm, Sweden), then processed and exported through Tobii Pro Lab 1.102.16417 software [47]. Due to the high dimensionality and multivariate characteristics of eye-tracking data, meticulous data processing and advanced statistical analysis techniques were utilized to ensure precision and reliability of the findings.

To evaluate participants’ reading task outcomes, written responses to post-reading questions were analyzed, producing two scores: recall (R) and perceived novelty (PN). The Recall score represents the total textual and visual information extracted from participants’ responses to the first question. The perceived novelty score captures participants’ perception of novelty regarding the scenic spot based on the second question. Table 2 illustrates examples of recall and perceived novelty score calculations. For example, participant #P9 referenced five sentences from the brochure’s textual and visual content (e.g., “Hongcun is a famous ancient cultural village of Huizhou” and “The Wang Family Ancestral Hall looks really gorgeous”), earning a Recall score of 5. For perceived novelty, the participant identified four novel aspects, yielding a PN score of 4. Researchers C.Z. and Y.M. independently coded and cross-checked all responses. In this study, the Cohen’s kappa for R was 0.92, and the Cohen’s kappa for PN was 0.91. Discrepancies were resolved through discussions with additional researchers to maintain consistency.

Data were analyzed statistically using SPSS for Windows v.26.0 (IBM, Armonk, NY, USA). Quantitative data, including travel motivation, Number of Fixations (NOF), and Reading Time (RT), were imported into SPSS. Descriptive statistics, such as mean (M) and standard deviation (SD), were calculated for travel motivation and eye-tracking metrics across each AOI. Independent sample t-tests compared data between Group 1 (G1) and Group 2 (G2) to identify significant differences, addressing RQ1. Correlation analyses were conducted to explore relationships among age, reading task outcomes, and eye-tracking metrics, addressing RQ2. A significance level of *p* < 0.05 was set as the threshold for statistical significance in all analyses.

To address RQ3, Lag Sequential Analysis (LSA) was performed using GSEQ 5.1 (Mangold International GmbH, Arnstorf, Germany) [17]. Scan path results were converted into fixation transitions between AOIs and analyzed for z-values and transition probabilities across the six AOIs. These metrics facilitated comparisons of visual behavior transition patterns between G1 and G2 [15]. In the LSA results, a z-value greater than 1.96 was deemed statistically significant.

## 3. Results

### 3.1. T-Test Results

The fixation count, fixation duration, and post-reading scores for recall (R) and perceived novelty (PN) were compared between younger and elder participants while viewing the brochure (Table 3). The results revealed no significant difference in recall scores between the two groups (*p* > 0.05). However, the PN scores of elder participants (M = 4.62, SD = 0.921) were significantly higher than those of younger participants (M = 3.59, SD = 1.118), t(46) = −3.402, *p* = 0.001.

**Figure 4:**
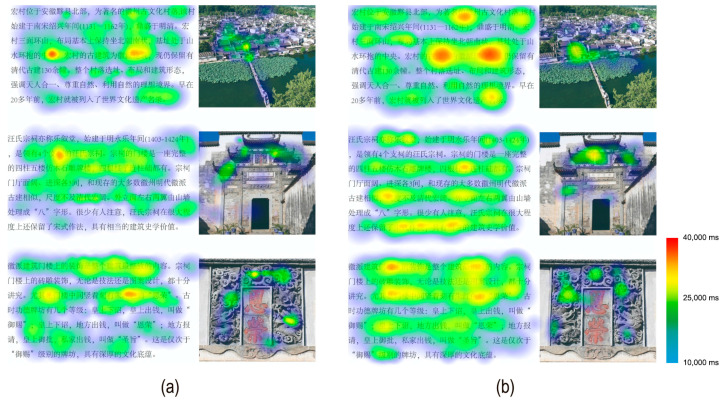
Heat map based on fixation duration: (**a**) younger group (**b**) elder group. (The English translation of the stimuli is shown in Figure 2 and Appendix A).

Eye movement data were analyzed by categorizing viewing areas into image regions (AOI 1 + AOI 3 + AOI 5) and text regions (AOI 2 + AOI 4 + AOI 6), with fixation count and fixation duration calculated for each category and their totals. Results showed no significant differences in fixation count or fixation duration within image regions between younger and elder participants (*p* > 0.05). However, elder participants demonstrated significantly higher fixation counts [t(46) = −3.767, *p* = 0.001] and fixation durations [t(46) = −4.957, *p* < 0.001] within text regions compared to younger participants. Additionally, elder participants exhibited significantly higher total fixation counts [t(46) = −3.474, *p* = 0.002] and total fixation durations [t(46) = −4.907, *p* < 0.001]. Figure 4 shows the heat map based on fixation duration.

### 3.2. Correlation Analysis Results

Correlations were analyzed between recall (R) and visual attention metrics, perceived novelty (PN) and visual attention metrics, and R and PN (Table 4). The results showed no significant correlations between R and any visual attention metrics (*p* > 0.05). However, PN demonstrated significant positive correlations with fixation duration on text (r = 0.301, *p* = 0.038) and total fixation duration (r = 0.356, *p* = 0.013).

### 3.3. Lag Sequential Analysis Results

To compare the fixation transition patterns of younger and elder participants, each fixation location was considered an event, and each transition between fixations was considered an action. Lag Sequential Analysis (LSA) was performed. Table 5 displays the z-values from the LSA results for fixation transitions in both groups. A z-value exceeding 1.96 indicates a significant fixation transition, representing a shift from the AOI in the first column to the AOI in the first row.

**Figure 5:**
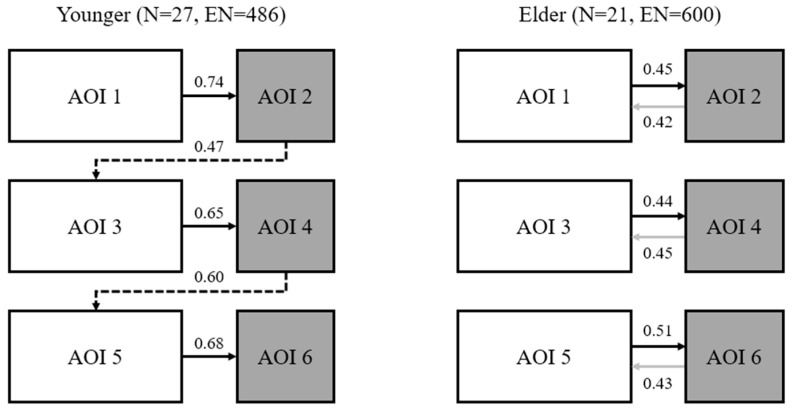
Fixation transition patterns of two groups.

To enable a clearer comparison of fixation transition patterns between younger and elder participants, Figure 5 was generated. In this figure, N denotes the number of participants in each group, and EN represents the total number of events (transitions). Significant fixation transitions are depicted with arrows, where arrow direction indicates transition direction, and associated numbers represent action probability. Solid black arrows indicate transitions common to both groups, dashed black arrows represent transitions unique to younger participants, and solid gray arrows show transitions unique to elder participants. The results suggest that, although the elder group included fewer participants than the younger group, they demonstrated more fixation transition behaviors. Younger participants’ fixation transition patterns adhered to a left-to-right and top-to-bottom reading sequence. In contrast, elder participants exhibited greater interaction between text and images, with bidirectional arrows connecting text and image AOIs across all three related levels.

## 4. Discussion

### 4.1. Reading Performance and Visual Attention of Younger and Elder Participants

This study identified significant differences in visual behavior and perception between younger and elder potential tourists when reading brochures. Overall, elder participants perform better in perceived novelty and allocate more visual attention to the text regions. However, there were no significant differences between them in recall scores and visual attention to images. These findings suggest that elder participants allocate more cognitive resources to processing textual information, while younger participants may depend more on holistic perception of image-based information.

These results are supported by the Dual-Task Theory [48] and Cognitive Load Theory [49], which propose that individuals process information in different ways depending on cognitive resources available. Elder participants, who tend to have higher cognitive load when processing text due to age-related changes in memory and attention, likely spend more time and attention on textual details, indicating deeper cognitive engagement [50]. On the other hand, younger participants may engage in more efficient visual scanning strategies, as they rely on less cognitive load to process visual information, favoring a more holistic understanding of images. This pattern aligns with the Information Processing Theory, which suggests that individuals of different age groups have distinct processing capacities and strategies, especially when faced with different types of stimuli [34].

Moreover, the higher perceived novelty among elder participants can be understood through Visual Attention Theory, which emphasizes how individuals process novel or unfamiliar information with more focused attention [51]. Elder participants’ higher perceived novelty may reflect their deeper cognitive engagement with textual details, which they perceive as more informative and unique. In contrast, younger participants’ preference for image-based comprehension aligns with findings in visual marketing research that highlight the importance of visual elements in attracting attention and promoting engagement [3].

In light of these theories, this study contributes to the understanding of how age-related differences shape visual cognitive strategies in information processing. Specifically, it highlights that elder individuals are more likely to engage in top-down processing, focusing on detailed, verbal information, while younger individuals tend to use bottom-up processing, focusing on visual and sensory features [52,53]. These cognitive strategies influence how information is processed and perceived in different formats, such as text versus images.

Compared to prior research, these findings build upon the work of Chen and Shoemaker [9] regarding the impact of age on tourism decision-making and information processing. The results further indicate that elder participants’ in-depth processing of textual information may be associated with their higher perceived novelty. This contrasts with the image-based comprehension of younger participants, which aligns with prior studies emphasizing the role of visual elements in marketing strategies [3]. However, this study also underscores the unique value of textual information in enhancing perceived novelty among elder participants, a factor seldom addressed in previous research.

Based on these findings, tourism brochure design could be optimized by emphasizing the richness and cultural depth of textual information to enhance perceived novelty among elder tourists while prioritizing the appeal and aesthetic coherence of visual elements for younger tourists. Future research could investigate whether similar visual information processing patterns occur across diverse cultural backgrounds, thereby validating the universality of these findings.

### 4.2. Correlation Between Reading Performance and Visual Attention

This study examined the relationship between reading task performance and visual attention, finding no significant correlation between recall (R) and visual attention metrics. This suggests that potential tourists’ memory retention may not solely rely on the distribution of their visual attention. Instead, recall might be more influenced by background knowledge or information encoding strategies than by the intensity or frequency of fixations. This aligns with the Cognitive Load Theory, which posits that cognitive resources and strategies for encoding information can impact memory retention more than the frequency of visual attention or fixations [49].

However, perceived novelty (PN) showed significant positive correlations with fixation duration in text regions and total fixation duration. This suggests that extended attention to textual information may enhance participants’ perception of novelty. These findings highlight the importance of in-depth processing of textual information in novelty perception and provide indirect support for Involvement Theory, which asserts that higher levels of engagement enhance information processing quality [34]. According to Involvement Theory, when individuals are more engaged with a task, they devote greater cognitive resources, which enhances the depth of processing and improves outcomes such as novelty perception. This theory explains why extended attention to textual information led to greater perceived novelty among participants.

This result further expands Zacks’ [21] conceptual framework on event perception and memory, highlighting the distinctive role of perceived novelty in visual information processing. While prior studies primarily emphasized the contribution of image-based information to novelty perception [3], this study demonstrates that the depth of textual information processing also plays a pivotal role in novelty perception. This finding may be associated with elder participants’ preference for detailed information and greater cognitive investment. Dual-Task Theory also provides insight into this observation, suggesting that older adults may allocate more cognitive resources to processing detailed information, particularly textual content, which leads to more in-depth processing and heightened novelty perception [48].

From a practical perspective, these findings offer actionable guidance for optimizing the design of tourism promotional materials. When promoting architectural heritage sites, designers should prioritize the quality and presentation of textual information, providing detailed and culturally rich descriptions to enhance perceived novelty among potential tourists. Furthermore, given that novelty positively influences tourists’ behavioral intentions, such design strategies may indirectly encourage actual visitation. Future research could delve into the specific mechanisms by which various content types affect novelty perception, such as experimentally manipulating the complexity and richness of textual and visual content to uncover their dynamic effects on novelty perception. Additionally, comparing different cultural contexts could help determine whether similar visual information processing patterns occur across various age groups in diverse cultural settings.

### 4.3. Difference in Fixation Transition Patterns

This study utilized Lag Sequential Analysis to investigate the fixation transition patterns of younger and elder potential tourists when reading brochures. The results showed that, despite the smaller sample size of the elder group, their visual transitions were more frequent and exhibited stronger interactions between text and images. This phenomenon may indicate a more integrative strategy adopted by elder participants, wherein frequent associations between text and images contribute to forming a more comprehensive understanding. In contrast, the younger group displayed a more linear fixation transition pattern, predominantly following a left-to-right and top-to-bottom reading sequence, indicating a preference for a direct scanning strategy in information processing. These findings are consistent with the principles of Dual-Task Theory, suggesting that elder participants allocate more cognitive resources to integrating different types of information, leading to more complex visual processing strategies [17,48]. This is further reflected in the transition probabilities: younger participants have a higher probability of shifting their gaze from the text on the left to the image on the right compared to older participants, which holds true across all three sets of text–image information. This suggests that younger participants prefer order and lack attention to the connections between pieces of information [54]. In contrast, older participants are more skilled at constructing mental representations of both text and images, reading the text while observing the visual information, actively searching for connections between them to form a comprehensive understanding [55]. After observing the image, there is more than a 40% chance that the next fixation will occur in the corresponding text area. While images have advantages in terms of intuitiveness and vividness, text provides more detailed and expansive information [56]. The combination of the information provided by both facilitates the generation of profound thoughts or strong emotions, which is beneficial for increasing tourism intention [57].

From a theoretical perspective, these findings further reinforce the depth of information processing theory, highlighting age-related differences in visual attention allocation. According to Involvement Theory, elder participants, possibly due to greater cognitive maturity or experience, appear more inclined to integrate cross-modal information (text and images) to enhance their comprehension of brochure content. This observation aligns with Leask and Barron’s [10] findings on age-related characteristics in information processing and extends the understanding of elder participants’ dynamic traits in visual information processing. Furthermore, these results challenge Castillo-Manzano and López-Valpuesta’s [8] claim that younger individuals are more sensitive to visual stimuli, suggesting that different reading tasks or visual contexts may impose varying demands on visual behavior, especially in complex visual environments like brochures.

Practically, these findings suggest that promotional materials targeting elder tourists should strengthen the association between text and image content, for example, by incorporating paired text–image explanations, to facilitate cross-modal information integration and enhance content comprehension. For younger tourists, emphasizing the attractiveness and visual fluidity of images may be more effective in rapidly capturing their interest. These recommendations are supported by Involvement Theory, which asserts that individuals with higher engagement (such as older participants) benefit from richer, more integrated content [33]. Future research could investigate the cultural or task-specific dependencies of these visual behavior patterns to validate the generalizability of these findings across diverse cultural contexts or task types.

### 4.4. Implications for Brochure Design

The findings of this study offer actionable recommendations for designing tourism brochures tailored to different age demographics, enhancing engagement and comprehension.

For Older Adults: Focus on Textual Clarity. Older participants in the study showed greater engagement with textual content, suggesting that brochures for older adults should prioritize larger font sizes, clear text–image alignment, and simple layouts. The use of high-contrast text and adequate line spacing can improve readability. Image–text pairing should be used to enhance comprehension, with brief and meaningful descriptions.

For Younger Adults: Visual Appeal and Interaction. Younger participants preferred a more visual approach, favoring image-dominant layouts. To engage this group, brochures should emphasize vibrant visuals, concise text, and interactive features like QR codes or augmented reality elements. Modern fonts and short paragraphs will also appeal to younger adults.

General Recommendations: Cohesion and Accessibility. For both groups, brochures should have a consistent layout with a clear hierarchical structure for easy navigation. Strong contrast between text and background is important, but the level of contrast should vary for each group. Clear calls to action should be included to guide readers toward the next steps.

By implementing these strategies, tourism marketers can create brochures that better serve the needs of both younger and older tourists, improving user experience and engagement.

### 4.5. Limitations and Furture Research

Despite the rigorous design of this study, several limitations persist. The sample size was relatively small, comprising only 48 participants divided into two age groups. This limitation may reduce statistical power and limit the generalizability of the findings, as a small sample might not adequately capture subtle variations within a broader population. Additionally, the sample was primarily sourced from travel agencies, which may introduce potential sampling bias. Travel agency clients may differ from general tourists in key factors such as travel frequency, type of travel experiences, and familiarity with marketing materials, which could affect how they interact with brochures. Future research should aim to recruit a more diverse sample from different settings to enhance the external validity and generalizability of the results. The visual stimuli consisted exclusively of static images and text from brochures, omitting multimedia or dynamic visual content. This may not fully capture the appeal and processing mechanisms of modern tourism marketing materials, such as videos or interactive brochures, potentially underestimating the impact of dynamic information on visual behavior. Furthermore, the stimuli and experimental design were specific to a particular region, so the data obtained are only valid for interpreting the situation in that geographic area and cannot be easily applied to other regions or cultural contexts. Not only that, the use of static brochures and predefined AOIs, while suitable for controlled experiments, may limit the ecological validity. In real-world settings, digital and interactive tourism content (such as websites and mobile apps) engage users more through multimedia elements and real-time navigation. The static format used here may not fully reflect how tourists interact with these dynamic materials. Future research should explore how interactive formats (such as videos or 360-degree images) affect visual attention and information processing to more accurately represent real-world behaviors and enhance the ecological validity of the findings. Finally, this study relied on eye-tracking data as the primary metric for visual attention, without integrating additional cognitive or physiological measures (e.g., brain activity or emotional responses). This limitation may constrain a comprehensive understanding of the cognitive mechanisms underlying visual behavior and its complexity. Future research could attempt to explore tourist cognitive behaviors and strategies from multiple dimensions and delve deeper into the underlying causes.

## 5. Conclusions

This study investigated the reading behaviors of architectural heritage tourism brochures by combining eye-tracking technology with reading task analysis. It examined how potential tourists from different age groups allocate visual attention and how these patterns correlate with reading performance.

By comparing fixation counts, fixation durations, and visual transition paths between younger and elder groups, the findings revealed that although recall performance did not differ significantly between the groups, elder participants scored significantly higher in perceived novelty and exhibited greater visual attention to textual regions. Additionally, elder participants exhibited more frequent cross-modal transitions between text and images, while younger participants adhered to a more linear scanning pattern. Lag Sequential Analysis further illustrated the transition paths across different levels of visual information, including overall destinations, individual buildings, and decorative details. These findings enhance the understanding of visual information processing and reading task performance, offering scientific guidance for optimizing promotional material design and improving the effectiveness of architectural heritage site promotion.

This study makes a novel contribution by exploring the interaction between visual behavior and information processing, illuminating how potential tourists of different age groups perceive and process promotional materials. Theoretically, the findings expand research boundaries on age-related differences in information processing modes, demonstrating how potential tourists allocate visual attention and process multi-level information in visually rich materials. These results validate the applicability of information processing theory and Involvement Theory while enriching the theoretical framework linking perceived novelty and visual behavior.

Methodologically, this study innovatively integrated eye-tracking and Lag Sequential Analysis to investigate visual transition patterns, with a specific focus on the dynamic integration of cross-modal information (text and images). This multi-layered analytical approach offers new avenues for future research on visual behavior across diverse task contexts. Practically, the findings provide empirical evidence to optimize the design of tourism brochures. For elder tourists, emphasizing the interaction between text and images and enhancing cultural depth is recommended, whereas for younger tourists, focusing on visual appeal and linear information flow may more effectively capture their attention. These strategies effectively enhance the perception of promotional materials and foster behavioral intentions among tourists.

## Figures and Tables

**Table 1:** Eye movement indicators and explanations.

Indicators	Explanation
Fixation count	Indicates the number of times a respondent’s eye rests on an area or point of interest while looking at it.
Fixation duration (s)	Refers to the amount of time a respondent’s eyes produce sweeping activity within an area while looking at that area.

**Table 2:** An example of the coding of a participant’s (P9) reading task outcomes.

	Recall (R)	Perceived Novelty (PN)
Response	…Hongcun is a famous ancient cultural village of Huizhou… (P9A1)	…It’s amazing that Hongcun has been so well-preserved for over 800 years… (P9B1)
	…The layout of the whole village emphasizes harmony between humans and nature… (P9A2)	…Hongcun isn’t just about architectural heritage; from an aerial view, you can see its beautiful natural environment too… (P9B2)
	…The Hui-style ancient buildings have a strong history… (P9A3)	…It’s incredible that such a high-standard gate tower has survived intact—truly rare… (P9B3)
	…The Wang Family Ancestral Hall looks really gorgeous… (P9A4)	…The carvings are so intricate; I find them absolutely stunning… (P9B4)
	…The arch gate of the Wang Family Ancestral Hall is very unique, with intricate ornamentations… (P9A5)	
Scores	5	4

**Table 3:** Outcomes of reading performance and visual attention.

Variable	Younger			Elder			t	*p*
	N	M	SD	N	M	SD		
R	27	4.30	1.103	21	4.62	0.973	−1.058	0.296
PN	27	3.59	1.118	21	4.62	0.921	−3.402	0.001
FC-image	27	149.67	37.858	21	151.24	37.660	−0.143	0.887
FC-text	27	416.81	86.537	21	537.86	125.934	−3.767	0.001
FC-total	27	566.48	88.225	21	689.10	141.807	−3.474	0.002
FD-image (s)	27	44.49	8.097	21	46.71	11.173	−0.800	0.428
FD-text (s)	27	127.46	23.023	21	163.93	27.954	−4.957	<0.001
FD-total (s)	27	171.95	24.379	21	210.64	30.277	−4.907	<0.001

**Table 4:** Correlation results between reading performance and visual attention.

Variable		R	PN	FC-Image	FC-Text	FC-Total	FD-Image	FD-Text	FD-Total
R	r	\	0.108	0.013	0.126	0.121	−0.043	0.182	0.158
	*p*	\	0.464	0.928	0.395	0.411	0.773	0.216	0.283
PN	r	0.108	\	0.227	0.232	0.283	0.259	0.301	0.356
	*p*	0.464	\	0.120	0.113	0.052	0.076	0.038	0.013

**Table 5:** z-values from LSA results for fixation transitions of two groups.

Variable		AOI 1	AOI 2	AOI 3	AOI 4	AOI 5	AOI 6
AOI 1	younger	0	10.91	−0.52	−3.53	−3.46	−3.07
	elder	0	6.82	−2.82	−1.12	−1.22	−1.55
AOI 2	younger	−0.78	0	7.43	−0.87	−1.62	−3.87
	elder	7.77	0	−0.17	−1.22	−2.03	−3.66
AOI 3	younger	0.63	−2.84	0	8.83	−2.56	−4.15
	elder	−2.79	0.19	0	5.17	−2.73	−0.38
AOI 4	younger	0.7	−3.07	−2.9	0	7.18	−2.54
	elder	−2.08	−2.44	6.35	0	−0.13	−1.97
AOI 5	younger	−1.21	−2.64	−4.04	−4.21	0	11.91
	elder	−0.61	−2.59	−3.59	−1.08	0	7.93
AOI 6	younger	0.85	0.18	0.32	−0.1	−0.83	0
	elder	−2.32	−1.72	−0.33	−1.87	6.2	0

## Data Availability

The data for this study are publicly available on the Github website—https://github.com/Zeno5577/Text-Image-and-Transition.git (accessed on 3 January 2025).

## Appendix A

Translation of stimuli

Hongcun, located in the northern part of Yi County, Anhui Province, is a renowned ancient cultural village of Huizhou. The village was founded during the Shaoxing period of the Southern Song Dynasty (1131–1162) and flourished during the Ming and Qing Dynasties. Surrounded by mountains on three sides, the layout of Hongcun predominantly faces south, with its site nestled in the heart of a landscape embraced by mountains and water. The ancient buildings in Hongcun are representative of Hui-style architecture, with over 130 preserved structures from the Qing Dynasty. The village’s site selection, layout, and architectural forms emphasize the ideal of harmony between humans and nature, respecting and utilizing natural surroundings. Over two decades ago, Hongcun was inscribed on the UNESCO World Heritage List.

The Wang Family Ancestral Hall, also known as Lexu Hall, was built during the Yongle period of the Ming Dynasty (1403–1424). It is the main ancestral hall of the Wang family, with four branch ancestral halls under its jurisdiction. The hall’s entrance gate is a complete four-post, five-story stone memorial archway designed to resemble wooden architecture, with all four pillars and their bases preserved. The entrance hall is three bays wide and three bays deep, similar to most Ming Dynasty Hui-style buildings in Huizhou, though not as expansive as those from the Qing Dynasty. The exterior facade features an “八”-shaped gable design on both sides. Notably, the Wang Family Ancestral Hall still retains many Song-style construction methods, giving it considerable value in the study of architectural history.

The ornamental features on the gate of Hui-style buildings are among the most intricate elements of the architecture. The brick carvings on the ancestral hall’s gate are exquisitely crafted, with meticulous techniques and intricate patterns. In particular, the central part of the gate is adorned with a vertically engraved, bright red “思荣” (“Thinking of Honor”). In ancient times, honorary arches were classified into several levels: “Imperial Bestowal”, funded by the emperor and issued by decree; “Grace and Honor”, issued by decree but funded by local authorities; and “Imperial Mandate”, where the emperor approved a local request for a privately funded arch. The one in the Wang Family Ancestral Hall is of the “Imperial Mandate” level, second only to “Imperial Bestowal”, reflecting its profound cultural significance.
